# Contribution of the respiratory network to rhythm and motor output revealed by modulation of GIRK channels, somatostatin and neurokinin-1 receptors

**DOI:** 10.1038/srep32707

**Published:** 2016-09-07

**Authors:** Gaspard Montandon, Hattie Liu, Richard L. Horner

**Affiliations:** 1 Departments of Medicine and Physiology, University of Toronto, Toronto, Canada.

## Abstract

Breathing is generated by a respiratory network in the brainstem. At its core, a population of neurons expressing neurokinin-1 receptors (NK1R) and the peptide somatostatin (SST) form the preBötzinger Complex (preBötC), a site essential for the generation of breathing. PreBötC interneurons generate rhythm and follower neurons shape motor outputs by activating upper airway respiratory muscles. Since NK1R-expressing preBötC neurons are preferentially inhibited by μ-opioid receptors via activation of GIRK channels, NK1R stimulation may also involve GIRK channels. Hence, we identify the contribution of GIRK channels to rhythm, motor output and respiratory modulation by NK1Rs and SST. In adult rats, GIRK channels were identified in NK1R-expressing preBötC cells. Their activation decreased breathing rate and genioglossus muscle activity, an important upper airway muscle. NK1R activation increased rhythmic breathing and genioglossus muscle activity in wild-type mice, but not in mice lacking GIRK2 subunits (GIRK2^−/−^). Conversely, SST decreased rhythmic breathing via SST_2_ receptors, reduced genioglossus muscle activity likely through SST_4_ receptors, but did not involve GIRK channels. In summary, NK1R stimulation of rhythm and motor output involved GIRK channels, whereas SST inhibited rhythm and motor output via two SST receptor subtypes, therefore revealing separate circuits mediating rhythm and motor output.

Breathing is an autonomic behaviour generated by a complex respiratory network in the brainstem. At its core is the preBötzinger Complex (preBötC), a population of neurons generating breathing^1^ by rhythmically activating respiratory neurons. In the preBötC, a population of interneurons generate rhythmic activity and other populations of follower neurons shape motor outputs and drive the neural circuits that activate the diaphragm during inspiration. PreBötC neurons also project to premotor hypoglossal neurons that drive hypoglossal motoneurons and upper airway muscle activity during inspiration^2^,^3^,^4^,^5^. PreBötC neurons regulate the amplitude of hypoglossal motor output because progressive, but not selective, destruction of inspiratory preBötC neurons decreases hypoglossal motor output in addition to affecting rhythmic breathing^6^. The functional roles and the identity of the preBötC neurons regulating rhythmic breathing and upper airway muscle activity have not been described.

PreBötC neurons express various G-protein-coupled receptors (GPCR) that are critical to maintaining breathing. A subpopulation of preBötC neurons expressing excitatory neurokinin-1 receptors (NK-1R) are necessary to generate breathing and their destruction impairs rhythmic breathing^7^. Another subpopulation of preBötC neurons contain somatostatin (SST)^8^, an inhibitory peptide that reduces neuronal activity by activating cognate SST receptors^9^. SST inhibits rhythmic breathing^10^ and inhibition of SST-expressing preBötC cells abolishes respiratory activity^11^, but the types of SST receptors involved are unclear^12^. Importantly, rhythmogenic preBötC interneurons are derived from a set of precursors that express the homeobox gene Dbx1^4^. Interestingly, Dbx1-expressing preBötC neurons also co-expressed SST_2A_ receptors^9^ and regulate hypoglossal motor output^3^. In addition, almost all NK-1R-expressing preBötC cells co-express SST^8^. SST-expressing preBötC neurons project to hypoglossal premotor areas^5^ and may therefore regulate upper airway muscle activity. In addition to SST and NK-1Rs, μ-opioid receptors (MOR) are present in preBötC neurons^13^ and their activation inhibits rhythmic breathing and upper muscle activity *in vivo*^14^ and *in vitro*^15^. Although the roles of MOR have been characterized^14^,^16^, the functional roles of NK-1R, SST, and SST receptors in regulating rhythmic breathing and upper airway muscle activity, especially *in vivo*, are unclear.

NK-1R and MOR are GPCRs that modulate neuronal activity through various pathways including calcium channels, adenylate cyclase, and/or G-protein-gated inwardly-rectifying potassium (GIRK) channels. In the preBötC, MORs inhibit rhythmic breathing through potassium channels^16^, and we recently identified that GIRK channels contribute to MOR respiratory slowing^17^. In nucleus basalis neurons, GIRK channels contribute to neuronal excitation by the endogenous NK-1R agonist substance P^18^. In the preBötC region, MORs reduce rhythmic breathing by preferentially inhibiting NK-1R-expressing preBötC neurons^14^ which suggests that NK-1Rs and MORs may involve similar pathways. Here, we propose that GIRK channels contribute to excitation of rhythmic breathing by NK-1Rs. Although MORs, SST, and SST_2A_ receptors are co-expressed in NK-1R-expressing preBötC cells^9^, activation of SST receptors inhibits glutamate release by activating potassium leak current or by inhibiting voltage-dependent calcium current^19^. We therefore suggest that the mechanisms underlying excitation by NK-1R and inhibition by MOR may differ from those regulating SST inhibition.

To better understand the circuitry generating rhythmic breathing and motor outputs, we aimed to establish the functional roles of GIRK-expressing preBötC neurons in mediating rhythmic breathing and hypoglossal motor activity, as well as excitation by NK-1Rs and inhibition by SST in adult rodents *in vivo*. Here, we propose a functional framework identifying the roles of GIRK channels, NK-1Rs, and SST receptors in rhythmic breathing and motor output.

## Methods

### Experimental animals

All procedures were performed in accordance with the recommendations of the Canadian Council on Animal Care, and were approved by the University of Toronto Animal Care Committee. 33 adult male Wistar rats (250–350 g, Charles River, Saint-Constant, Quebec, Canada), 8 wild-type mice (4 females and 4 males), and 9 mice lacking the GIRK2 subunit (GIRK2^−/−^, 5 females and 4 males) were used for physiological recordings (body weight: 40–50 g). The GIRK channel subunits GIRK1, GIRK2, and GIRK3 form 4 types of tetramers and the absence of GIRK2 subunits eliminates 3 out of 4 tetramers in the brain^20^,^21^. The generation of GIRK2^−/−^ mice was described previously^20^. Mice and rats were housed with free access to food and water under a 12-hour light 12-hour dark cycle (lights on at 7 am).

### Microperfusion and recordings in anesthetized adult rodents

In anesthetized adult male rats, we used reverse-microdialysis to unilaterally microperfuse selected agents into the preBötC. The experimental procedures were as described previously^14^,^22^. Briefly, we recorded activities of diaphragm and genioglossus muscles in isoflurane-anesthetized (2–2.5%), tracheotomised and spontaneously breathing (50% oxygen gas mixture, balance nitrogen) adult rats. Diaphragm muscle activity was recorded using stainless steel bipolar electrodes positioned and sutured on the right side of the crural diaphragm. Genioglossus muscle activity was recorded using two stainless steel needles inserted into the muscle. Electromyography signals were amplified (CWE Inc., Ardmore, Pennsylvania, USA), band-pass filtered (100–1000 Hz), integrated and digitized at a sampling rate of 1000 Hz using CED acquisition system and Spike v6 software (Cambridge Electronic Design Limited, Cambridge, England). Rats were kept warm with a heating pad during the experiments. Using a dorsal approach, a microdialysis probe (CX-I-12-01, Eicom, Kyoto, Japan) of 200 μm diameter and 1 mm diffusing membrane length was inserted into the preBötC using a stereotaxic frame and micromanipulator (ASI Instruments, Warren, Michigan, USA). The probe was placed 12.2 mm posterior, 2 mm lateral, and 10.5 mm ventral to bregma according to standard rat brain atlas^23^. We used three criteria to accurately target the preBötC, to position the probe and to confirm its location as described previously^14^,^22^. (i) When the probe was inserted in the brain, genioglossus muscle activity showed a reduction of about 30% as it reached the vicinity of the preBötC. (ii) Microperfusion of the μ-opioid receptor agonist DAMGO 5 μM decreased respiratory rate by approximately 50%^14^. (iii) Post-mortem histology was used to confirm the probe location in the preBötC using anatomical markers such as the semi-compact division of nucleus ambiguus, the caudal part of the facial nucleus, and immunohistochemistry of NK-1R. In rats, we used the caudal part of the facial nucleus as a reference to identify the brain section located 11.6 mm posterior to Bregma. In mice, we used the caudal part of the facial nucleus to identify the brain section 6.5 mm posterior to Bregma. Once the coronal section where the probe was located was identified, we used the nucleus ambiguus to determine the medial-lateral and dorsal ventral coordinates. We used these three anatomical and functional criteria and experience from our previous studies to confirm that the probes were positioned in the region of the preBötC. On rare occasions (1/20 experiments), the probes damaged the preBötC and respiratory rhythm was irregular and unstable. In such an event, we did not continue the experiment. Fresh artificial cerebrospinal fluid (aCSF) was made according to the following composition (in mM): NaCl (125), KCl (3), KH2PO4 (1), CaCl2 (2), MgSO4 (1), NaHCO3 (25), and glucose (30). pH was adjusted to 7.4 by bubbling CO_2_ into aCSF. A microdialysis probe was perfused with aCSF and baseline levels of the physiological variables were recorded for least 30 min followed by 120 min of recordings during perfusion of the selected agents. Breathing rate, diaphragm muscle amplitude and genioglossus muscle amplitude were averaged over a 10 min period at the end of 30 min of baseline, and over a 10 min period after 30 min of drug perfusion. We applied different GPCR agonists and antagonists and channel modulators. To activate GIRK channels, we added flupirtine (200 μM) or ML-297 (50 or 200 μM) to the aCSF perfusing the preBötC as previously described (Montandon *et al.*^17^). We also perfused the NK1-R agonist GR73632 (50 μM), the SST_2_ receptor antagonist CYN-154806 (20 μM)^24^, the peptide somatostatin (200 μM)^25^, the SST_4_ receptor agonist (NNC 26–9100) and the GIRK channel blocker Tertiapin Q (1 μM). All drugs were obtained from Tocris (Minneapolis, Minnesota, USA).

In anesthetized (isoflurane, 1.5–2%), spontaneously breathing (50% oxygen, balance nitrogen) adult mice, we also used reverse microdialysis to perfuse agents into the preBötC of wild-type and GIRK2^−/−^ animals. We recorded diaphragm and genioglossus muscle activities using a similar approach to the rats and as previously described^17^. The mice were also kept warm with a heating pad. We inserted the microdialysis probe into the brainstem 6.7 mm posterior, 1.2 mm lateral, and 5.7 mm ventral to bregma according to standard mouse brain atlas^26^. For wild-type or GIRK2^−/−^ mice, baseline levels were recorded for at least 30 min followed by 120 min of recordings. The anatomical and functional criteria defined in rats were also used for experiments in mice. Because of the random production of GIRK2^−/−^ knockout animals during breeding, we did not randomize wild-type and GIRK2^−/−^ mice. However, we alternated recordings from wild-type and knockout mice to avoid order effects or experimental conditions that may affect one group temporarily. To avoid experimenter bias, similar standard procedures, timelines for dosing, and automated analyses were used for both animal groups. Breathing rate, diaphragm muscle amplitude and genioglossus muscle amplitude were averaged over a 10 min period at the end of 30 min of baseline and over a 10 min period after 30 min of drug perfusion.

### Construction of correlation maps

We constructed correlation maps to relate the location of the intervention sites with the resultant effect on respiratory activity as previously described and validated^14^,^22^. The rationale for the construction of correlation maps is that for a locus of effect of drugs (ML-297) at any particular brainstem site, the latency for the drug to diffuse through the tissue and to progressively change respiratory activity will vary as a function of the distance of the probe from the effective site. We first determined the locations of the intervention (perfusion) sites by using anatomical markers and standard brain maps^23^. To determine the anterior-posterior coordinates, we used the caudal end of the facial nucleus as the reference point which is located 11.6 mm posterior to bregma. The dorsal-ventral and medial-lateral coordinates were defined using the nucleus ambiguus and standard brain maps. For each animal, we then calculated the latencies to a 10% change in respiratory rate or the percentage of reduction after 30 min of drug perfusion. For every possible sets of coordinates, we then calculated the distances from the perfusion sites to the corresponding set of coordinates and correlated these distances with the latencies for the effects on respiratory activities or percentage of rate change. We first presented the correlation between distances from preBötC (coordinates −12.3 mm anterior, 2 mm medial, and 10 mm ventral to bregma) to perfusion sites and latencies or rate changes. Then, using Matlab 12 software (Mathworks, Natick, MA), the correlation coefficients (0 < R < 1) were calculated for every set of coordinates and plotted as colour pixels (blue to red) in a standard brain map 12.3 mm posterior to bregma^23^.

### Immunohistochemistry in rats

Animals were euthanized with isoflurane, perfused with phosphate buffer (pH = 7.4), and then paraformaldehyde (PFA, 4%). Brain were harvested and fixed for 4 hours in PFA, and then transferred to sodium citrate (1%) overnight for antigen retrieval. Brains were then boiled for 4 min in sodium citrate and immersed in sucrose solution (30%) overnight. Brains were frozen at −20 °C and coronal sections (50 μm) were cut in a cryostat. Sections were then incubated with rabbit anti-GIRK2 (1:500, Alomone Labs, Jerusalem, Israel) and goat anti-NK-1 (1:500, Sigma-Aldrich, Oakville, ON, Canada) antibodies, and with donkey anti-rabbit IgG Alexa 488 (1:200, Jackson ImmunoResearch, West Grove, PA, USA) and anti-goat IgG Alexa 594 (1:200, Jackson ImmunoResearch) secondary antibodies. Sections were then incubated with DAPI (NucBlue, Life Technologies Inc., Burlington, ON, Canada) mounted on slides, and then visualized with an epifluorescence microscope (AxioScan, Leica Microsystems Inc., Concord, ON, Canada). We focused our immunofluorescence on the 300 μm-extension of the medulla around preBötC neurons (six consecutive 50 μm sections starting 150 μm caudal to the preBötC). As anatomical markers, we use the caudal part of the hypoglossal motor nucleus, the inferior olive, the nucleus ambiguus, and the facial nucleus. We counted numbers of immunopositive cells within a circle of 1 mm of diameter centered in the preBötC. To identify the location of the preBötC, we performed additional immunohistochemistry for NK-1Rs^7^ using the Avidin-Biotin Complex method and chromogen diaminobenzidine staining. The antibodies used were rabbit anti-NK1R (1:1000, AB-N04, Advanced Targeting Systems, San Diego, CA, USA) and donkey anti-rabbit IgG (1:100, Jackson ImmunoResearch).

### Statistical Analysis

In Figures, group data are presented as mean ± standard error mean (S.E.M.) and individual data are also displayed. We used S.E.M. to improve visibility in graphs. In Results section, data are presented as mean ± standard deviation (SD). For the studies in rats, we used 1-way ANOVAs with the repeated factor being treatments with aCSF or drugs. For the studies in mice, we tested for group differences using 2-way repeated measure ANOVA tests, with a factor being genotype (i.e. wild-type or GIRK2^−/−^) and the repeated factor being drug treatments (i.e. aCSF or drugs of choice). If the ANOVA was statistically significant, an All Pairwise Multiple Comparison Procedure (Holm-Sidak tests) was then used to determine significant differences between conditions (aCSF/baseline versus drug). When statistical tests were not significant, the power of the test was indicated to avoid interpreting non-significant tests as evidence of absence of effects. All hypothesis tests are two-tailed with the level of significance set at *P* < *0.05*. All tests were performed with SigmaPlot version 11 (Systat Software Inc, San Jose, CA, USA).

## Results

### GIRK2 subunit expression in the preBötC

**Figure 1:**
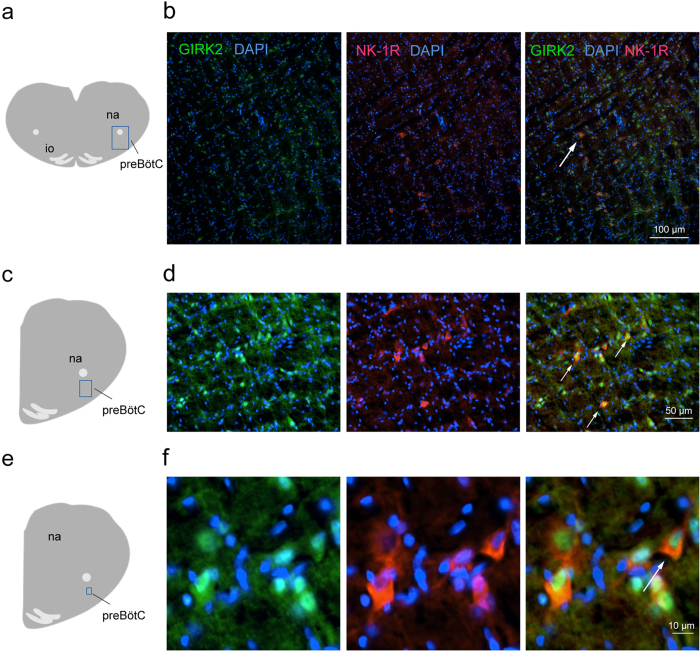
Expression of GIRK2 subunits and neurokinin-1 receptors in preBötC neurons of adult rats. In the region of the preBötC ventral to the nucleus ambiguus (**a**), GIRK2 subunits were expressed in somas (GIRK2 *green,* DAPI, *blue*, **b**). Neurokinin-1 receptors were expressed in the preBötC region (*red*, **c**). All cells expressing NK-1Rs also expressed GIRK2 subunits (white arrow, **d**). Magnification showed perinuclear expression of GIRK2 subunits in cells of the preBötC region (**e**,**f**). NK-1Rs were found in somas and co-expressed with GIRK2 subunits. Results are representative of three biological replicates. Scale bars 100, 50, and 10 μm for upper, middle and lower panels respectively. na, nucleus ambiguus. io, inferior olive.

In neurons, the GIRK2 subunit is an integral subunit found in 3 out of 4 GIRK tetramers^21^. The GIRK2 subunit is expressed in the region of the preBötC and modulates rhythmic breathing in rodents^17^. To identify the types of preBötC cells expressing GIRK channels, we visualized the expression of GIRK2 subunits and NK-1Rs using immunohistochemistry of brainstem sections from adult rats (n = 3). GIRK2 subunits were present in the somas of small cells (diameter < 100 μm) in the preBötC (Fig. 1a), ventral to the nucleus ambiguus (anterior-posterior position: 12.3 mm posterior to bregma) and caudal to the Bötzinger Complex (Fig. 1b). Combined with the nuclear marker DAPI, we determined that GIRK2 subunits were expressed in 22.6 ± 4.2% of cells in the preBötC (n = 3, Fig. 1c,d). Cells with somas expressing NK-1R all co-expressed GIRK2 subunits (Fig. 1e,f). We counted that 15.1 ± 4.6% of GIRK2-positive cells co-expressed NK-1Rs (n = 3). These results showed that a relatively small proportion of cells in the preBötC region expressed GIRK2 subunits and NK-1Rs.

### Functional role of GIRK channels in the preBötC

**Figure 2:**
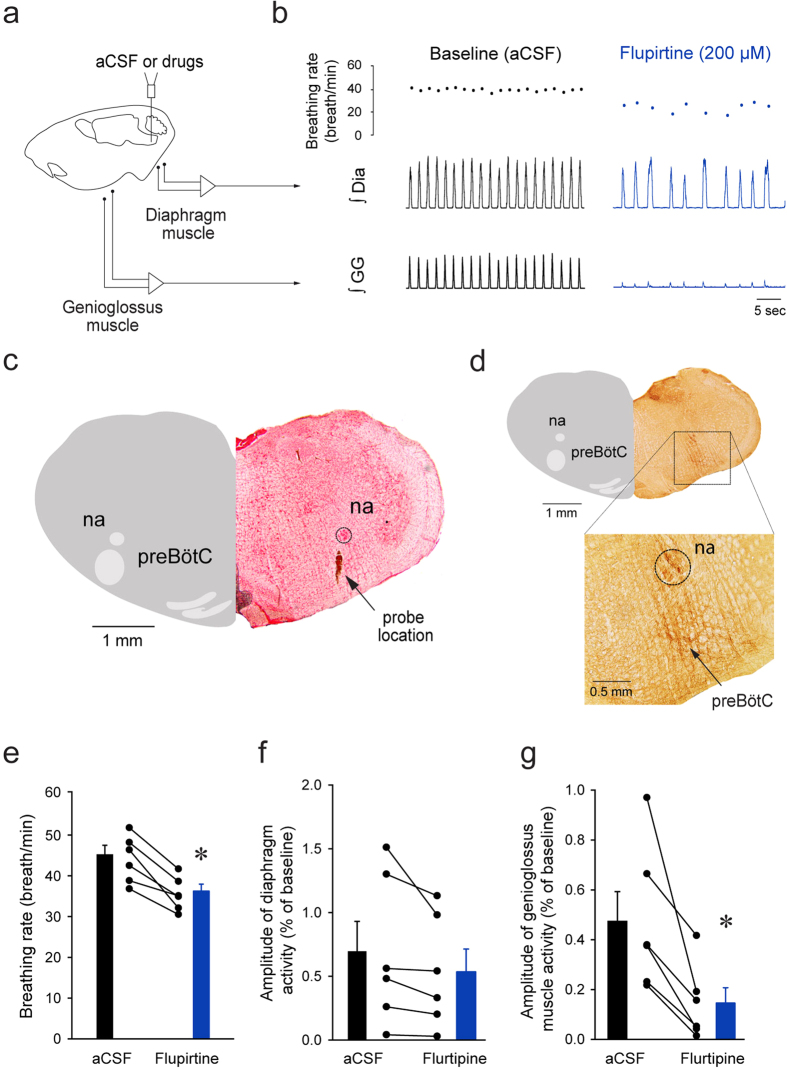
Activation of GIRK channels with flupirtine at the preBötC decreased rhythmic breathing and genioglossus muscle activity in anesthetized rats. Microperfusion (**a**) of flupirtine (200 μM) in the preBötC region reduced breathing rate and genioglossus muscle amplitude, but not diaphragm amplitude (**b**). Dots indicate breathing rate for each breath. The microperfusion probe was positioned in the region of the preBötC (**c**) identified by high expression of NK-1Rs (**d**). Ventral to the nucleus ambiguus, NK-1Rs were highly expressed and identified the preBötC region (see magnification). Flupirtine significantly reduced breathing rate (n = 6, **e**), but not diaphragm muscle amplitude (**f**). Flupirtine also significantly diminished genioglossus muscle amplitude (**g**). *Indicate mean values significantly different from aCSF/baseline with *P* < *0.05*. Data are indicated as means ± S.E.M and as individual values for each animal. aCSF, artificial cerebrospinal fluid. Dia, diaphragm muscle. GG, genioglossus muscle.

Activation of GIRK channels using flupirtine inhibits rhythmic breathing in rats and wild-type mice, but not in GIRK2 subunit knockout mice (GIRK2^−/−^)^17^, suggesting that flupirtine preferentially activates GIRK channels. To further establish the functional role of GIRK channels in modulating upper airway muscle activity, we replicated previous experiments^17^ by micro-perfusing flupirtine into the preBötC of anesthetized adult rats using reverse microdialysis while recording diaphragm and genioglossus muscle activity (Fig. 2a) as previously described^17^. In a representative rat, flupirtine (200 μM) decreased breathing rate when perfused into the region of the preBötC as previously demonstrated^17^. Interestingly, flupirtine also decreased genioglossus muscle activity (Fig. 2b) when perfused in the preBötC region (Fig. 2c) as identified by the high expression NK-1Rs, ventral to the nucleus ambiguus (Fig. 2d)^7^,^14^. Flupirtine (200 μM) significantly decreased breathing rate by 19.1 ± 7.4% (*P* = *0.002*, n = 6, Fig. 2e), similar to our previous study^17^. Diaphragm muscle amplitude was not significantly affected by flupirtine (*P* = *0.105*, n = 6, Fig. 2f). Importantly, flupirtine also significantly decreased genioglossus muscle amplitude by 72.5 ± 22.6% (*P* = *0.017*, n = 6, Fig. 2g).

### Selective activation of GIRK channels in the preBötC

**Figure 3:**
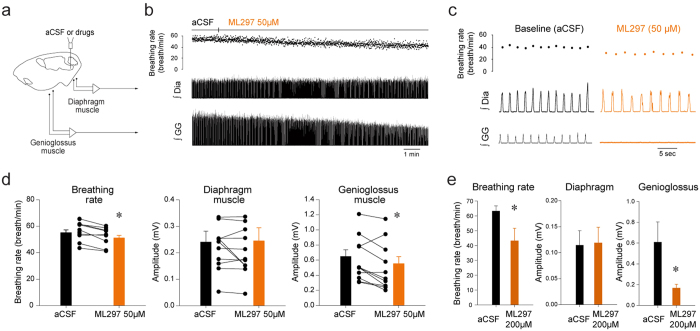
Activation of GIRK channels with ML297 applied in the preBötC region decreased rhythmic breathing and genioglossus muscle amplitude in anesthetized rats. Microperfusion (**a**) the selective GIRK channel activator ML297 (50 μM) in the preBötC region reduced breathing rate and genioglossus muscle amplitude, but not diaphragm amplitude (**b**,**c**). Dots indicate breathing rate for each breath. ML297 (50 μM) significantly reduced breathing rate (n = 11, **d**), but not diaphragm muscle amplitude (**f**). ML297 also significantly diminished genioglossus muscle amplitude. ML297 at higher concentration (200 μM strongly reduced breathing rate and genioglossus muscle amplitude, but not diaphragm amplitude (**e**). *Indicate mean values significantly different from aCSF/baseline with *P* < *0.05*. Data are indicated as means ± S.E.M and as individual values for each animal. aCSF, artificial cerebrospinal fluid. Dia, diaphragm muscle. GG, genioglossus muscle.

In addition to modulate GIRK channels, flupirtine activates voltage-sensitive potassium channels, indirectly antagonizes NMDA receptors and modulates GABA_A_ receptors^27^,^28^. To selectively target GIRK channels, we micro-perfused the GIRK channel activator ML297^29^. Microperfusion of ML297 (50 μM) into the preBötC region (Fig. 3a) decreased breathing rate and genioglossus muscle amplitude, but not diaphragm amplitude (Fig. 3b,c). ML297 (50 μM) significantly decreased breathing rate (Fig. 3d, *P* = *0.005*, n = 11) and genioglossus muscle amplitude (*P* = *0.016*, n = 11), but did not change diaphragm muscle amplitude (*P* = *0.898*, n = 11). In a separate set of experiments, ML297 perfused at higher concentration (200 μM) substantially decreased breathing rate (Fig. 3e, *P* = *0.049*, n = 4) and genioglossus muscle (*P* = *0.045*, n = 4). Overall, these results identified that activation of GIRK channels at the preBötC level decreased breathing rate and genioglossus muscle activity.

### Focal activation of GIRK channels in the preBötC

**Figure 4:**
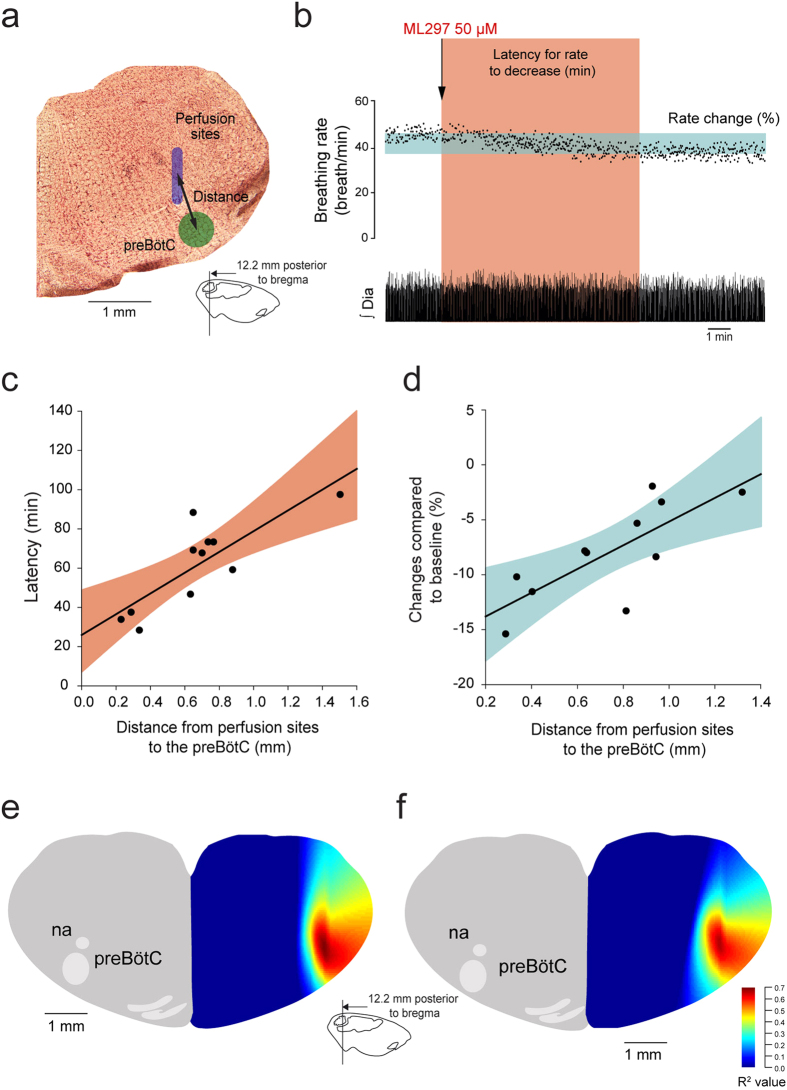
Relationships between proximity of perfusion sites to preBötC and changes in breathing rate induced by ML297 in anesthetized rats. For each experiment where ML297 was perfused in the preBötC region, the distance from the perfusion site to the center of the preBötC was calculated (**a**). Also, the latency for ML297 (50 μM) to decrease breathing rate by 10% (red shade, **b**) or the change in rate after 30 min of perfusion (blue shade) was estimated. The correlation between latencies and distances for n = 11 animals was then calculated. Significant and positive correlations between distances and latencies (R = 0.818, *P* = *0.002*, n = 11, **c**), and distances and rate changes (R = 0.766, *P* = *0.006*, n = 11, **d**) were found suggesting that ML297 induced faster and more pronounced reductions in rate when perfused close to the preBötC. Correlation maps indicated regions of the brainstem where correlation between distances and latencies (**e**) or changes (**f**) were higher (red hot spots), and these hotspots correspond to the preBötC.

To better identify the region of the ventrolateral medulla where ML297 activates GIRK channels, we quantified the relationship between the proximity of the perfusion site to the preBötC and the latency for ML297 to change breathing. If ML297 activates GIRK channels specifically at the preBötC, then the latency for the drug to perfuse through tissue and affect breathing should be shorter when the perfusion is performed close to the preBötC than when it is performed away from it. Based on this concept, we related the distance from the perfusion site to the center of the preBötC (Fig. 4a) with the latency for ML-297 to decrease breathing rate by 10% (Fig. 4b) as previously validated^14^,^22^. For each experiment, we calculated the latency for ML297 to decrease breathing rate by 10% and the distance from perfusion site to the center of the preBötC. The center of the preBötC used in this study was 12.3 mm posterior, 2 mm lateral, and 10 mm ventral to Bregma^23^. We then found a significant correlation between distances and latencies (R = 0.818, *P* = *0.002*, n = 11, Fig. 4c). Similarly we related the distances with the degree of reduction induced by ML297 and found a positive significant correlation between distances and rate changes (R = 0.766, *P* = *0.006*, n = 11, Fig. 4d). These correlations suggest that close perfusion of ML297 to the preBötC quickly and substantially decreased breathing rate compared to perfusions away from it. As examples in experiments where perfusion occurred close to the preBötC (distance < 0.4 mm), ML297 reduced breathing rate by more than 15% (Fig. 4d). To eliminate the possibility that there were other sites in the medulla that were equally sensitive to ML297 than the preBötC, we calculated the correlation coefficients for all the possible sites in the medulla and created color maps representing the area of the medulla where ML297 induced a fast and substantial decrease in breathing rate. In the region of the preBötC, correlation coefficients were above 0.6 (red spot) suggesting high correlation between latencies and distances (Fig. 4e). A similar correlation map was generated for distances and rate changes and identified a similar region with high correlation (Fig. 4f). These data therefore identified the preBötC as an area of the medulla highly sensitive to ML297 and suggest that selective activation of GIRK channels in the preBötC decreased breathing rate.

### NK-1R modulates rhythmic breathing and genioglossus muscle activity through GIRK channels

**Figure 5:**
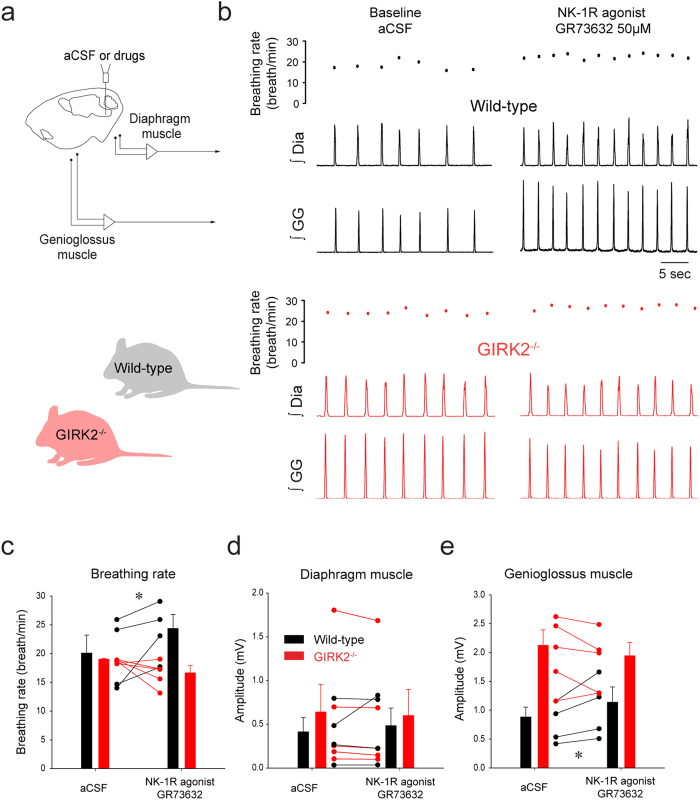
Activation of NK-1Rs increased rhythmic breathing and genioglossus muscle amplitude in the wild-type but not in the GIRK2^−/−^ mice. Microperfusion of the NK-1R agonist GR73632 (50 μM) into the preBötC (**a**) significantly increased breathing rate in wild-type mice (**b**), but not in GIRK2^−/−^ mice. Dots indicate breathing rate for each breath. Mean data showed that breathing rate was increased in wild-type mice (n = 4), but not GIRK2^−/−^ mice (n = 5, **c**). Diaphragm muscle amplitude was not changed by GR73632 either in wild-type nor in GIRK2^−/−^ mice (**d**). Genioglossus muscle amplitude was increased by GR73632 in wild-type, but not in GIRK2^−/−^ mice (**e**). *Indicate mean values significantly different from aCSF/baseline with *P* < *0.05*. Data are indicated as means ± S.E.M and as individual values for each animal.

Destruction of NK-1R-expressing neurons severely disrupts rhythmic breathing^7^, but the functional roles of NK-1R in mediating rhythmic breathing and genioglossus muscle activity *in vivo* remains unclear^25^,^30^. We therefore microperfused the NK-1R agonist GR73632 into the preBötC of anesthetized wild-type mice (Fig. 5a). GR73632 (50 μM) applied to the preBötC increased rhythmic breathing in the wild-type mice by 25.7 ± 15.6% (*P* = *0.048*, n = 4, Fig. 5b,c). Diaphragm muscle amplitude was not significantly changed by GR73632 (*P* = *0.885*, n = 4, Fig. 5b,d). GR73632 significantly increased genioglossus muscle amplitude by 26.4 ± 9.8% in wild-type mice (*P* = *0.035,* Fig. 5b,e).

NK-1R-expressing preBötC neurons are selectively inhibited by MOR *in vitro*^14^ and respiratory inhibition by MOR depends on GIRK channels^17^. Since NK-1R excitation inhibits GIRK channels in the nucleus basalis^18^, excitation of preBötC neurons by NK-1R may also involve GIRK channels. In mice lacking GIRK2 subunits, we applied GR73632 (50 μM) to the preBötC and breathing rate was unchanged unlike the stimulation elicited in the wild-type mice (2-way ANOVA, mouse genotype x drugs, *P* = *0.018*, Fig. 5b,c). The two-way ANOVA (drugs x genotype) was not significant for diaphragm amplitude (*P* = *0.228*, Fig. 5d). Similar to breathing rate, genioglossus muscle amplitude was increased by GR73632 in the wild-type mice but not in the GIRK2 mice (2-way ANOVA, mouse genotype x drugs, *P* = *0.016,* Fig. 5b,e). These data identify that GIRK channels significantly contribute to the excitatory effect of NK-1R on respiratory activity.

### SST and respiratory activity *in vivo*

**Figure 6:**
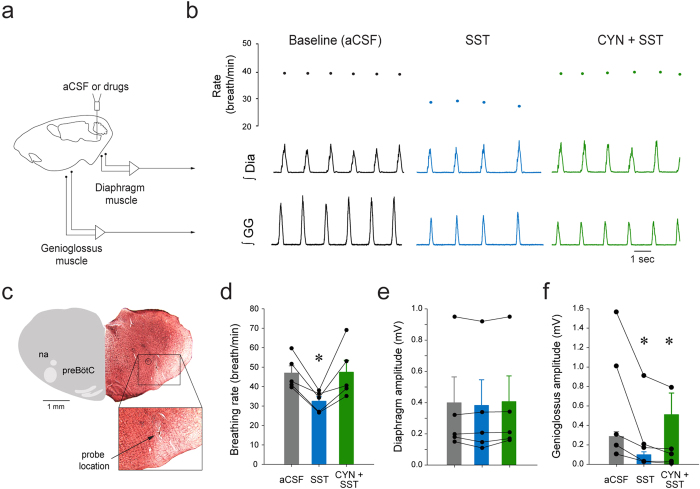
Modulation of rhythmic breathing and genioglossus muscle amplitude by SST and SST_2_ receptors in anesthetized rats. Microperfusion of SST (200 μM) into the preBötC (**a**) depressed rhythmic breathing and genioglossus muscle amplitude (**b**), reduction that were blocked by the SST_2_ receptor antagonist CYN-154806 (20 μM, **b**). Dots indicate breathing rate for each breath. The microperfusion probe was located ventral to the nucleus ambiguus in the preBötC region (**c**). SST decreased breathing rate (n = 5, **d**) and CYN-154806 blocked the inhibition by SST. Diaphragm muscle amplitude was not changed by SST or CYN-154806 (**e**). SST also diminished significantly genioglossus muscle amplitude and this decrease was not blocked by the SST_2_ receptor antagonist (**f**). *Indicate mean values significantly different from aCSF/baseline with *P* < *0.05*. Data are indicated as means ± S.E.M and as individual values for each animal. SST, somatostatin. CYN, the SST_2A_ antagonist CYN-154806.

The inhibitory peptide SST is co-expressed with NK-1R in a large proportion of preBötC^8^. Inhibition of SST-expressing neurons abolishes rhythmic breathing in rats^11^ and application of SST in the preBötC of adult rats decreases breathing rate^31^. Although SST-expressing neurons project to hypoglossal premotor neurons^5^, their functional role in modulating genioglossus muscle amplitude has not been determined. In anesthetised rats, microperfusion of SST (200 μM) into the preBötC (Fig. 6a) decreased respiratory rate and genioglossus muscle activity (Fig. 6b). Histology confirmed that the probe was located ventral to the nucleus ambiguus and in the region of the preBötC (Fig. 6c). In 5 rats, SST slowed respiratory rate by 27.3 ± 8.8% (*P* = *0.014*, n = 5, Fig. 6d). This inhibition was blocked by concomitant microperfusion of the SST_2_ receptor antagonist CYN-154806 (100 μM). Diaphragm muscle amplitude was not significantly changed by SST or CYN-154806 at the preBötC (*P* = *0.105*, n = 5, Fig. 6e). SST decreased genioglossus muscle amplitude by 38.9 ± 25.3% (*P* = *0.038*, n = 5, Fig. 6f), but this inhibition was unaffected by CYN-154806 at the preBötC (*P* = *0.732*, n = 5). These results identified that SST inhibited breathing rate via SST_2_ receptors, whereas it decreased genioglossus muscle amplitude via another non-SST_2_ receptors.

### SST_4_ receptors and genioglossus muscle activity

**Figure 7:**
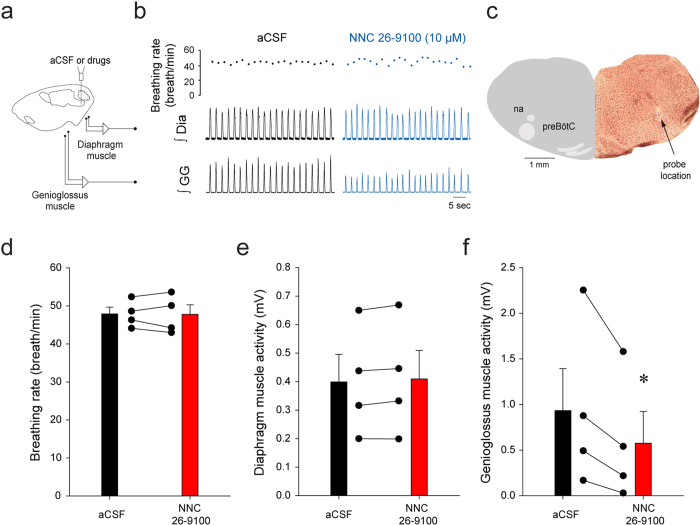
SST_4_ receptor activation at the preBötC did not change rhythmic breathing but decreased genioglossus muscle amplitude in anesthetized rats. Microperfusion of the SST_4_ receptor antagonist NNC 26–9100 (10 μM) into the preBötC of anaesthetized rats (**a**) decreased genioglossus muscle activity but not breathing rate and diaphragm amplitude (**b**). The microperfusion probe was located in the preBötC ventral to the nucleus ambiguus (**c**). NNC 26–9100 (10 μM) into the preBötC did not significantly change breathing rate (**d**) or diaphragm muscle amplitude (**e**), but significantly reduced genioglossus muscle amplitude (n = 4, **f**). *Indicate mean values significantly different from aCSF/baseline with *P* < *0.05*. Data are indicated as means ± S.E.M and as individual values for each animal.

To identify the type of SST receptors regulating inhibition of genioglossus muscle activity by SST, we perfused the SST_4_ receptor agonist NNC 26–9100^32^ into the preBötC (Fig. 7a). There is currently no available antagonist that is selective for SST_4_ receptors. NNC 26–9100 (10 μM) did not change breathing rate (Fig. 7b–d, *P* = *0.902*) or diaphragm muscle amplitude (Fig. 7e, *P* = *0.092*), but significantly reduced genioglossus muscle activity by 54.9 ± 27.7% (*P* = *0.048*, Fig. 7f). These results showed that activation of SST_4_ receptors in the preBötC inhibited genioglossus muscle activity, without affecting breathing rate and diaphragm amplitude.

### SST and GIRK channels

**Figure 8:**
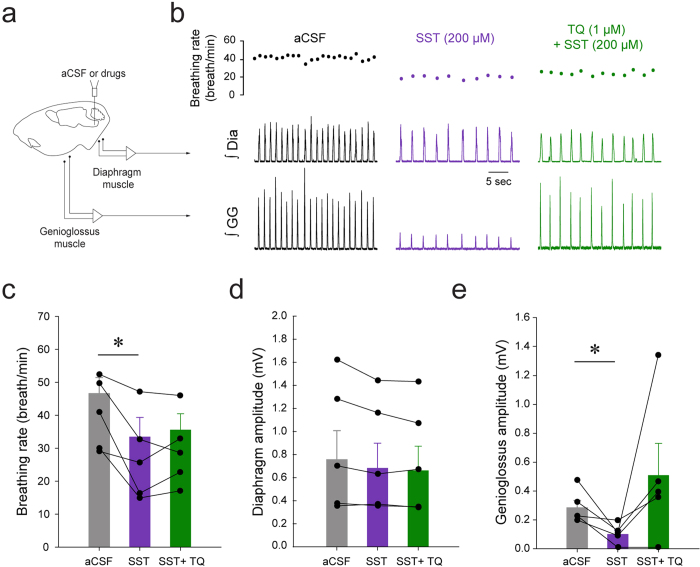
Inhibition of genioglossus muscle amplitude by SST, but not inhibition of breathing rate, was blocked by the GIRK channel blocker Tertiapin Q. Microperfusion of SST (200 μM) into the preBötC of anaesthetized rats (**a**) decreased breathing rate and genioglossus muscle activity, but only genioglossus muscle amplitude was reversed by Tertiapin Q (**b**). SST significantly decreased breathing rate, but Tertiapin Q was not able to block this inhibition (n = 5, **c**). SST significantly decreased genioglossus muscle amplitude and this depression was reversed by Tertiapin Q (1 μM). *Indicate mean values significantly different from aCSF/baseline with *P* < *0.05*. Data are indicated as means ± S.E.M and as individual values for each animal.

SST, through binding to its cognate receptors, inhibits glutamate release by activating potassium leak current or inhibition of voltage-dependent calcium current^19^. Because SST, MOR, and NK-1R are co-expressed in the same cells, these GPCRs may share similar mechanisms to modulate neuronal activity. We therefore tested whether GIRK channels regulate inhibition of respiratory activity by SST by blocking in the preBötC GIRK channels using the GIRK channel blocker Tertiapin Q (1 μM). Perfusion of SST to the preBötC (Fig. 8a) decreased breathing rate and genioglossus muscle amplitude, but did not affect diaphragm amplitude (Fig. 8b). In 5 rats, SST significantly decreased breathing rate (Fig. 8c, *P* = *0.022*, n = 5), an effect that was not blocked by Tertiapin Q (*P* = *0.578*, n = 5). Diaphragm amplitude was not affected by SST or Tertiapin Q (Fig. 8d, *P* = *0.055*, n = 5). Genioglossus muscle amplitude was not significantly changed by SST or SST + Tertiapin Q due to the high variability of muscle amplitude (Fig. 8e, *P* = *0.101*, n = 5).

**Figure 9:**
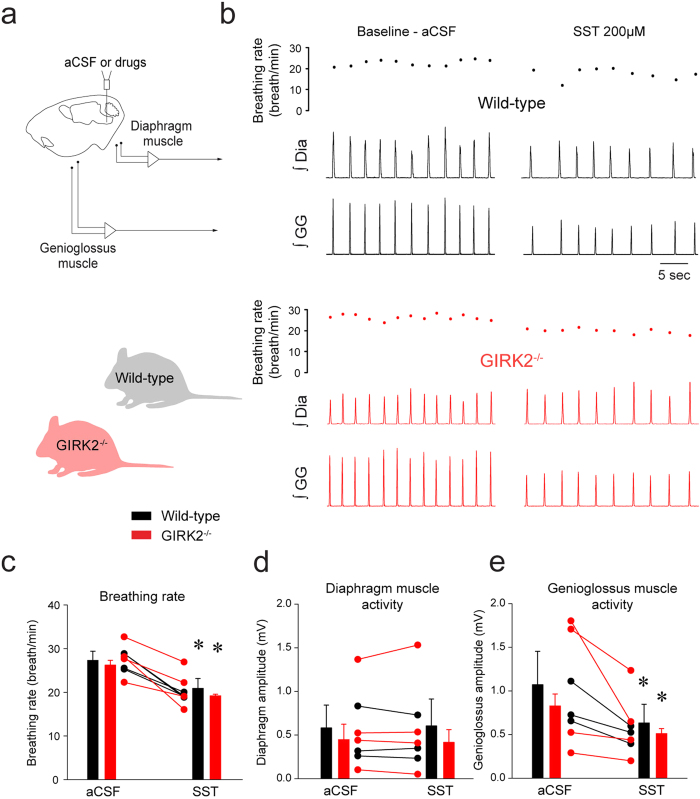
SST decreased rhythmic breathing and genioglossus muscle amplitude in the wild-type and GIRK2^−/−^ mice. Microperfusion of SST (200 μM) into the preBötC (**a**) significantly increased breathing rate in wild-type mice (**b**) and in GIRK2^−/−^ mice. Mean data showed that breathing rate was decreased wild-type (n = 4) and GIRK2^−/−^ mice (n = 4, **c**). Diaphragm muscle amplitude was not changed by SST either in wild-type nor in GIRK2^−/−^ mice (**d**). Genioglossus muscle amplitude was decreased by SST in wild-type, but not in GIRK2^−/−^ mice (**e**). *Indicate mean values significantly different from aCSF/baseline with *P* < *0.05*. Data are indicated as means ± S.E.M and as individual values for each animal.

To better assess the contribution of GIRK channels to inhibition of breathing by SST, we perfused SST in wild-type and GIRK2^−/−^ mice (Fig. 9a,b). In wild-type mice, microperfusion of SST into the preBötC decreased breathing rate by 23.3 ± 12.9% (*P* = *0.040*, n = 4, Fig. 9c). Diaphragm muscle activity was unchanged by SST (*P* = *0.668*, n = 4, Fig. 9d). SST did not decrease genioglossus muscle activity (*P* = *0.171*, n = 4, Fig. 9e). In GIRK2^−/−^ mice, SST decreased breathing rate by 26.5 ± 6.8% (*P* = *0.036*). Two-way ANOVA showed that there was no interaction between genotype x conditions (*P* = *0.824*, n = 4), therefore showing that SST decreased breathing rate in GIRK2^−/−^ mice. Diaphragm muscle activity was also not differently changed by SST in wild-type and GIRK2^−/−^ mice (*P* = *0.438*). SST also significantly decreased genioglossus muscle activity in GIRK2^−/−^ mice by 36.9 ± 9.4% (*P* = *0.021*) and there was no interaction between genotype and conditions (*P* = *0.698*). We also assessed whether breathing rate in wild-type and GIRK2^−/−^ differed in males and females. A 2-way ANOVA showed that females, males wild-type and GIRK2^−/−^ present similar breathing rate (genotype x sex, *P* = *0.648*, n = 8 males and n = 9 females). In summary, these results demonstrate that reduction of respiratory activity by SST was not dependent on GIRK channels.

## Discussion

Understanding the structural and functional organization of the neural circuits generating rhythmic breathing and upper airway muscle activity is essential to comprehend how breathing is generated. At the core of the respiratory network, preBötC neurons co-express NK-1Rs, SST, and MORs, and project to hypoglossal premotor areas^3^,^5^ driving the hypoglossal motor pools and genioglossus muscle activity. Although the identities of preBötC neurons essential for rhythmic breathing have been established, the functional roles of NK-1Rs and SST receptors in regulating rhythmic breathing and upper airway muscle activity are unknown. Here, we first established that NK-1R activation in the preBötC increases rhythmic breathing and genioglossus muscle activity. Conversely, SST reduces rhythmic breathing by activating SST_2_ receptors. Interestingly, SST_4_ receptor activation decreases genioglossus muscle activity, but not breathing rate, therefore suggesting that SST_4_ receptors may mediate the SST-induced changes in motor output. We further explored the underlying mechanisms mediating respiratory activity in the preBötC and identified that GIRK channels modulate NK-1R stimulation of rhythmic breathing and upper airway muscle activity, but not inhibition by SST.

### GIRK channels and preBötC

GIRK2 subunits, which contribute to forming 3 out of 4 types of GIRK channels, were expressed in the region of the preBötC. Importantly, a subpopulation of GIRK2-expressing neurons also expressed NK-1Rs. In mice, GIRK2 subunits were also found in the preBötC region^17^. Small fusiform preBötC interneurons express NK-1R and SST^8^ and are thought to be the site of respiratory rhythm generation in rodents^7^. Hence cells co-expressing GIRK2 and NK-1Rs may constitute a subpopulation of the small fusiform NK-1R/SST important for the generation of rhythmic breathing^33^.

### Functional role of GIRK channels

The anatomical location, the identity of the cells expressing GIRK2 subunits, and our previous data^17^ suggests that GIRK channels in preBötC cells are essential components of the neural circuit modulating breathing. To further test this concept we manipulated GIRK channels in the preBötC region of adult rats and measured the subsequent changes in respiratory activity. Our results showed that GIRK channel activation at the preBötC decreased rhythmic breathing, a result that was previously identified^17^. Importantly, we further identified that GIRK channels decreased genioglossus muscle activity. Flupirtine decreased rhythmic breathing and genioglossus muscle amplitude in wild-type, but not in GIRK2^−/−^ mice^17^, suggesting that flupirtine inhibited breathing selectively through GIRK channels *in vivo*. In summary, we identified that activation of GIRK channels in the preBötC region diminished respiratory rhythm, and most importantly, motor output.

### NK-1Rs and GIRK channels

Substance P, a peptide that binds to NK-1Rs, NK-2Rs, and NK-3Rs, increased phrenic nerve frequency in rabbits^25^ and rats^31^, but the NK-1R agonist GR73632 did not stimulate rhythmic breathing in rabbits^25^. These results are not consistent with the clear role of NK-1Rs in generating rhythmic breathing *in vivo*^7^,^34^, and our results showing that selective activation of NK-1Rs in the preBötC region increased rhythmic breathing. In addition to increase rhythmic breathing, NK-1R activation also increased genioglossus muscle activity. This result is consistent with NK-1R-expressing preBötC cells projecting directly to premotor hypoglossal neurons^33^. Similarly, application of substance P to the preBötC increased rhythmic breathing and hypoglossal motor output *in vitro*^16^. We then further explored the contribution of GIRK channels to NK-1R-related increase in respiratory activity. We identified that NK-1R activation did not significantly increase breathing rate and motor output in mice lacking GIRK2^−/−^ subunits compared to wild-type animals, a result suggesting that NK-1Rs stimulate neuronal activity via inhibition of GIRK channels, although the direct link between NK-1Rs and GIRK channels remains to be specifically demonstrated.

### SST receptors and respiratory activity

SST-expressing preBötC cells are essential for the generation of breathing in adult rats *in vivo*^11^ and application of SST to the medulla decreases rhythmic breathing^31^. PreBötC interneurons are thought to generate rhythmic breathing, but a sub-population of follower SST preBötC neurons also project to premotor hypoglossal neurons^33^. Rhythmogenic preBötC interneurons are derived from a set of precursors that express the homeobox gene Dbx1^4^. Interestingly, Dbx1-expressing preBötC neurons also co-expressed SST_2A_ receptors^9^ and regulate hypoglossal motor output^3^. The existence of subpopulations of SST preBötC neurons suggests that SST may play different functional roles in modulating respiratory rhythm and motor output. In the present study, SST into the preBötC decreased rhythmic breathing and genioglossus muscle activity. Interestingly, SST_2_ receptor blockade reversed the effect of SST on rhythmic breathing, but not on genioglossus muscle activity, a finding consistent with the role of SST_2A_ receptors *in vitro*^9^. On the other hand, SST_4_ receptor activation only decreased genioglossus muscle activity. Due to the lack of available selective SST_4_ receptor antagonist, the role of SST_4_ receptors in mediating SST-related reduction of genioglossus muscle activity cannot be tested. Furthermore, respiratory inhibition by SST was not mediated by GIRK channels suggesting that different mechanisms may mediate respiratory inhibitions by SST and MOR^17^. Overall, these results highlight two receptor subtypes underlying SST-mediated inhibition of respiratory network activity. SST at the preBötC inhibits rhythmic breathing via SST_2_ receptors, whereas SST may inhibit genioglossus muscle activity via SST_4_ receptors.

### NK-1R excitation and MOR inhibition

The relationship between MORs and NK-1Rs keeps the balance between nociception and analgesia, with activation of MORs inhibiting the release of neurotransmitters involved in nociception, therefore reducing neurotransmission related to pain^35^. Similarly, NK1-R-expressing preBötC neurons are preferentially inhibited by MOR agonists^14^. Activation of NK-1Rs increases rhythmic breathing and genioglossus muscle activity, whereas inhibition by MORs decreases them^14^,^15^. Importantly, GIRK channels contribute to respiratory inhibition by MORs^17^ and respiratory stimulation by NK-1Rs. Here we propose that GIRK channels in the preBötC maintain the balance between inhibitory and excitatory GPCRs and stabilize breathing (Montandon *et al.*^17^). When such balance is altered, for instance by opioid drugs, then breathing is depressed^36^,^37^. Likewise central apneas can occur following destruction of NK-1R-expressing preBötC cells in animals^34^ or in patients with multiple lateral sclerosis^38^. Overall, identification of GIRK channels as an important component of the respiratory circuitry that maintains stable breathing is central to understanding respiratory network function and dysfunction.

## Additional Information

**How to cite this article**: Montandon, G. *et al.* Contribution of the respiratory network to rhythm and motor output revealed by modulation of GIRK channels, somatostatin and neurokinin-1 receptors. *Sci. Rep.* **6**, 32707; doi: 10.1038/srep32707 (2016).
